# Supplementary dataset to self-learning training compared with instructor-led training in basic life support

**DOI:** 10.1016/j.dib.2019.104064

**Published:** 2019-05-26

**Authors:** Helene Bylow, Thomas Karlsson, Andreas Claesson, Margret Lepp, Jonny Lindqvist, Johan Herlitz

**Affiliations:** a Department of Molecular and Clinical Medicine, Institute of Medicine, Sahlgrenska Academy, University of Gothenburg, Gothenburg, Sweden; b Health Metrics Unit, Institute of Medicine, Sahlgrenska Academy, University of Gothenburg, Gothenburg, Sweden; c Department of Medicine, Centre for Resuscitation Science, Karolinska Institute, Stockholm, Sweden; d Institute of Health and Care Sciences, Sahlgrenska Academy, University of Gothenburg, Sweden, Østfold University College, Halden, Norway; e School of Nursing and Midwifery, Griffith University, Australia; f Centre of Registers Västra Götaland, Gothenburg, Sweden; g Prehospen-Centre of Prehospital Research, Faculty of Caring Science, Work Life and Social Welfare, University of Borås, Sweden

## Abstract

In this article, we present supplementary data to the article entitled “Self-learning training versus instructor-led training in basic life support: a cluster randomised trial” [1]. In three supplementary files, we present the informed consent of the included participants, the modified instrument to calculate the total score for practical skills called “the Cardiff Test of basic life support and automated external defibrillation” and the questionnaire to obtain background factors, theoretical knowledge, self-assessed knowledge and confidence and willingness to act, distributed directly after training and six months after training. The results of comparisons between “directly after intervention” and “six months after intervention”, for each training group separately, are presented in three tables. We also present two tables showing the reasons why the participants were not prepared to perform compressions and/or ventilations in the event of a sudden out-of-hospital cardiac arrest.

**Table:** Specification table

Subject area	Medicine
More specific subject area	Cardiology, cardiac arrest and education
Type of data	Supplementary files and tables with analysed and raw data
How data were acquired	Data were acquired from an intervention with education in basic life support (BLS) and automated external defibrillation (AED). Informed consent was required from each participant. A modified instrument called “the Cardiff Test of basic life support and automated external defibrillation” calculated the total score for practical skills. A questionnaire directly after training and six months after training acquired background factors such as characteristics and theoretical knowledge, self-assessed knowledge, confidence and willingness. A training manikin connected to a data program measured the variables. A video camera filmed the assessment and was used to control the direct observation of the assessment.
Data format	Analysed and raw data
Experimental factors	This experiment was a cluster randomised, controlled trial. The self-learning training in BLS constituted the experimental group and the instructor-led training in BLS constituted the control group. The primary outcome was the total score on the Cardiff Test of basic life support and automated external defibrillation for adherence to the BLS-AED algorithm.
Experimental features	Participants were enrolled from a BLS-AED research project. Effectiveness of self-learning training in BLS-AED was compared with instructor-led training in BLS-AED.
Data source location	Data were collected at 84 workplaces in the south of Sweden in 2014–2016. Data source location: Alingsås, Almenäs, Borgstena, Borås, Brotorp, Brämhult, Byttorp, Dalsjöfors, Eskilsryd, Falköping, Fristad, Frufällan, Gothenburg, Helsingborg, Hyberg, Kinna, Kyllared, Limmared, Malmö, Mölnlycke, Sandared, Sjöbo, Sjömarken, Skene, Skövde, Spånga, Tranemo, Trollhättan, Viared and Viskafors.
Data accessibility	The data are presented in this article.
Related research article	Bylow H, Karlsson T, Claesson A, Lepp M, Lindqvist J, Herlitz J. Self-learning training versus instructor-led training for basic life support: A cluster randomised trial. Resuscitation. 2019.

**Table:** 

**Value of the data** • The data provide information on the informed consent, valuable for scientific researchers. • The instrument called the Cardiff Test of basic life support and automated external defibrillation, modified to guidelines, was used to calculate the total score for practical skills and provide crucial information on the included variables. This can be valuable both for the interpretation of the data and for other experiments. • The data provide the variables in the questionnaire directly after intervention and six months after intervention for an insight into the self-assessed variables and for further investigations. • The data present changes from post-test to retention test, for each group separately, which may be useful for discussions, further insights and development of new experiments. • The data provide reasons given by the participants for unwillingness to perform CPR in an OHCA situation, which may be useful for discussions, further insights and development of new experiments.

## 1 Data

In this article, we present supplementary data to the article entitled “Self-learning training versus instructor-led training in basic life support: a cluster randomised trial”, where we compared the total score for practical skills and theoretical and self-assessed knowledge, confidence and willingness to act, between two training interventions in basic life support (BLS) with automated external defibrillation (AED) [1]. The training was based on the European Resuscitation Council (ERC) guidelines [2], [3]. Education with frequent training in BLS with AED may increase early high-quality cardiopulmonary resuscitation (CPR) with early defibrillation in the event of a sudden out-of-hospital cardiac arrest (OHCA) [4].

The data shared in this article contain supplementary files and tables and supplement the article by Bylow et al., 2019. Informed consent was required from each participant and is presented in this article in Supplementary file 1.

The instrument used to calculate the total score, the modified Cardiff Test of basic life support and external defibrillation, is presented in Supplementary file 2. Actions to evaluate education in BLS and the performance of CPR with an AED, definitions and scoring documents to assess the participants, were introduced as a statement on the uniform reporting of education in resuscitation [5], [6], in 2003. The Cardiff Test in this article was based on the previous statement and modified according to the European Resuscitation Council (ERC) guidelines [2], [3] and previous studies [5], [6], [7], [8], [9], [10], [11], [12] and was then tested in a pilot study [13].

The questionnaire used to collect background factors and the participants’ self-assessed theoretical knowledge, confidence and willingness to perform CPR with an AED in a real-life OHCA situation, directly after intervention and six months later, is presented in Supplementary file 3.

To supplement the cluster randomised, controlled trial [1], we present data from comparisons between post-test and retention test, for each training group, i.e. self-learning training and instructor-led training in BLS, separately, in this article. Table 1 presents analysed values from the Cardiff Test of basic life support and external defibrillation, Table 2 shows analysed values from individual variables and Table 3 presents analysed values from the self-assessed variables.

**Table 1:** The Cardiff Test of basic life support and external defibrillation – Changes from post-test directly after intervention to retention test six months after intervention (only those who participated at both occasions are included).

Variables	Self-learning training	p-value	Instructor-led training	p-value
	(n = 653)		(n = 521)	
	+/-a		+/-a	
Checks responsiveness—by talking				
Yes	2.1/5.1	0.04	1.3/3.3	0.06
Checks responsiveness—by shaking				
Yes	2.9/4.9	0.13	2.7/3.8	0.27
Opens airway—by head tilt and chin lift				
Perfect as instructed	15.5/26.5	0.009	12.3/32.2	0.0006
Checks breathing—by look, listen and feel				
Yes	8.4/11.2	0.20	3.5/16.3	0.0003
Call 112 or asks for call to 112				
Yes	5.5/2.9	0.14	1.3/6.0	0.004
Asks for an AED				
Yes	16.4/11.9	0.14	8.8/11.1	0.21
Starts CPR—compression/ventilation ratio				
30:2 (28–32:2)	6.6/20.4	<0.0001	4.4/23.2	<0.0001
Hand placement compressions				
Correct	12.3/16.8	0.08	16.9/14.6	0.53
Average compressions depth				
50–59 mm	21.4/16.2	0.06	20.2/16.1	0.15
Average compression rate				
100-120	18.1/16.1	0.45	14.6/16.3	0.48
Total compressions counted				
140-190	24.5/19.0	0.07	18.4/17.9	0.88
Average ventilation volume				
500–600 ml	6.9/7.4	0.88	7.9/7.7	0.94
Total ventilations counted				
8-12	19.0/21.1	0.42	10.7/23.2	0.007
Total hands-off time				
≤60 seconds	4.7/2.6	0.03	2.9/1.7	0.31
Switches on the AED				
Yes	0.6/0.0	1.00^b^	0.6/0.0	1.00^b^
Attaches electrode pads				
Both pads completely in areas	8.4/6.3	0.27	5.6/10.9	0.02
Checks safety				
Yes	15.6/21.4	0.27	7.5/31.5	0.0001
Delivers shock as directed by the AED				
Yes	1.1/0.2	0.07^b^	0.8/0.4	0.69^b^
Resumes CPR immediately after shock				
Yes	5.7/9.5	0.03	2.5/9.8	0.0003
Total score^c^				
Median	0	0.49	−2	<0.0001
25th,75th percentile	−4,3		−5,1	
Min, max	−18,31		−17,16	

Data collected from the Resusci Anne manikin, the PC SkillReporting System (Laerdal Medical, Stavanger, Norway) and direct observations.

Except for change in total score, where a generalized Wilcoxon Signed Rank for Paired Comparisons of Clustered Data was used, all comparisons were performed using Obuchowski's Extension of McNemar's Test for Clustered Data or, when percentages were very small, McNemar's exact test, without adjustment for clustering, was used. All tests are two-sided and p-values below 0.05 were considered statistically significant.

a Percentage of participants fulfilling the criteria at retention test six months after intervention but not at post-test directly after intervention (i.e. improvement)/percentage of participants fulfilling the criteria at post-test directly after intervention but not at retention test six months after intervention (i.e. impairment).

b McNemar's exact test used, without adjustment for clustering.

c Change in total score from post-test directly after intervention to retention test six months after intervention.

**Table 2:** Individual variables for quality of practical skills for cardiopulmonary resuscitation and external defibrillation (CPR-AED) – Changes from post-test directly after intervention to retention test six months after intervention (only those who participated at both occasions are included).

Variables	Self-learning training (n = 653)	p-value	Instructor-led Training (n = 521)	p-value
	a		a	
Correct compressions (%) (5/3)^b^				
median	0	0.81	0	0.89
25th,75th percentile	−23,21		−22,24	
Compressions with insufficient depth (%) (5/3)				
median	0	0.86	−0	0.53
25th,75th percentile	−13,11		−12,8	
Compressions with incorrect hand-position (%) (5/3)				
median	0	0.06	0	0.07
25th,75th percentile	−18,28		−17,27	
Compressions with incomplete release (%) (5/3)				
median	0		0	
25th,75th percentile	0,0		0,0	
>0 %^c^	9.4/13.9	0.03	9.5/14.7	0.04
Average compression depth (mm) (0/0)				
median	0	0.87	0	0.58
25th,75th percentile	−3,5		−3,4	
Average compression rate (per minute) (0/0)				
median	2	0.005	3	0.04
25th,75th percentile	−7,13		−7,11	
Correct ventilations (%) (5/3)				
median	0		0	
25th,75th percentile	−1,1		−1,1	
>0 %^c^	21.6/20.8	0.85	23.7/18.9	0.29
Average ventilation volume (ml) (0/0)				
median	−23	0.46	−150	0.03
25th,75th percentile	−329,249		−452,148	
Time to start of CPR (seconds) (0/0)				
median	−3	0.002	−1	0.28
25th,75th percentile	−11,5		−8,7	
Time to first shock (seconds) (4/2)				
median	−1	0.04	4	0.002
25th,75th percentile	−15,10		−8,14	

Data collected from the Resusci Anne manikin, the PC SkillReporting System (Laerdal Medical, Stavanger, Norway) and direct observations.

A generalized Wilcoxon Signed Rank for Paired Comparisons of Clustered Data was used for change in continuous variables and Obuchowski's Extension of McNemar's Test for Clustered Data for dichotomous variables. All tests are two-sided and p-values below 0.05 were considered statistically significant.

a Change from post-test directly after intervention to retention test six months after intervention.

b Number of participants where information was missing in the two training groups, respectively.

c Percentage of participants with >0% at retention test six months after intervention but not at post-test directly after intervention (i.e. improvement)/percentage of participants with >0% at post-test directly after intervention but not at retention test after six months (i.e. impairment).

**Table 3:** Participants self-assessed theoretical knowledge, confidence and willingness to act in a real-life sudden out-of-hospital cardiac arrest (OHCA) situation – Changes from post-test directly after intervention to retention test six months after intervention (only those who participated at both occasions are included).

Variables	Self-learning training (n = 653)	p-value	Instructor-led training (n = 521)	p-value
	+/-a		+/-a	
Self-assessed theoretical knowledge and practical skills to be able to perform compressions (157/84)^b^	1.8/0.6	0.14	1.4/1.1	0.81
Self-assessed theoretical knowledge and practical skills to be able to perform ventilations (157/92)	2.0/0.2	0.01	0.9/0.7	0.74
Self-assessed theoretical knowledge and practical skills to be able to use an AED (220/111)	6.7/2.8	0.10	2.2/2.4	0.89
Self-assessed confidence after training (69/56)	1.4/1.2	0.80	0.4/1.1	0.33
Self-assessed willingness to act if a relative suffers an OHCA (0/4)				
Would not dare or want to intervene	0.6/1.2	0.39^c^	0.2/0.2	1.00^c^
Would give ventilations only	0.5/0.3	1.00^c^	0.2/0.0	1.00^c^
Would give chest impressions only	1.1/2.1	0.25	1.9/0.8	0.12
Would give both chest compressions and ventilations	3.2/1.7	0.08	1.0/2.3	0.11
Self-assessed willingness to act if an unknown person suffers an OHCA (5/5)				
Would not dare or want to intervene	1.5/2.2	0.40	1.6/1.4	0.77
Would give ventilations only	0.2/0.3	1.00^c^	0.2/0.4	1.00^c^
Would give chest impressions only	9.9/6.8	0.07	12.4/6.8	0.002
Would give both chest compressions and ventilations	6.6/9.0	0.13	7.0/12.6	0.002
Theoretical knowledge of first action if cardiac arrest, i.e. call to 112 (14/6)	7.2/17.4	0.0004	5.6/23.5	<0.0001

All comparisons were performed using Obuchowski's Extension of McNemar's Test for Clustered Data or, when percentages were very small, McNemar's exact test, without adjustment for clustering, was used. All tests are two-sided and p-values below 0.05 were considered statistically significant.

a Percentage of participants with a Yes answer at retention test after six months but a No at post-test directly after intervention/percentage of participants with a Yes answer at post-test directly after intervention but a No at retention test after six months.

b Number of participants where information was missing in the two training groups, respectively.

c McNemar's exact test used, without adjustment for confounding.

In Table 4, we present reasons given by the participants for not being willing to perform compressions and/or ventilations in a sudden real-life out-of-hospital cardiac situation.

**Table 4:** Reasons for unwillingness to act in a real-life sudden out-of-hospital cardiac arrest (OHCA) situation, directly after intervention and six months after intervention.

	Directly after Self-learning training (n = 661)	Intervention Instructor-led training (n = 540)	Six months after Self-learning training (n = 670)	Intervention Instructor-led training (n = 561)
Self-assessed willingness to act if a relative suffers an OHCA				
Would not give chest compressions	N = 15	N = 2	N = 13	N = 4
Lack of knowledge	n = 6	n = 1	n = 2	n = 2
Afraid of hurting victim	n = 6	n = 2	n = 5	n = 3s
Afraid of transmittable/contagious disease	n = 2	n = 0	n = 0	n = 1
Other reasons^a^	n = 3	n = 1	n = 5	n = 1
Unknown reason	n = 1	n = 0	n = 2	n = 0
Would not give ventilations	N = 29	N = 11	N = 19	N = 17
Lack of knowledge	n = 8	n = 2	n = 4	n = 3
Afraid of hurting victim	n = 5	n = 3	n = 3	n = 6
Afraid of transmittable/contagious disease	n = 9	n = 4	n = 2	n = 8
Other reasons^b^	n = 8	n = 3	n = 9	n = 5
Unknown reason	n = 6	n = 4	n = 4	n = 3
Self-assessed willingness to act if an unknown person suffers an OHCA				
Would not give chest compressions	N = 32	N = 12	N = 28	N = 15
Lack of knowledge	n = 14	n = 2	n = 10	n = 6
Afraid of hurting victim	n = 12	n = 4	n = 13	n = 8
Afraid of transmittable/contagious disease	n = 7	n = 2	n = 6	n = 5
Do not want to touch a stranger	n = 3	n = 3	n = 5	n = 2
Other reasons^c^	n = 5	n = 4	n = 7	n = 3
Unknown reason	n = 2	n = 3	n = 0	n = 1
Would not give ventilations	N = 229	N = 154	N = 250	N = 187
Lack of knowledge	n = 15	n = 5	n = 12	n = 9
Afraid of hurting victim	n = 15	n = 9	n = 12	n = 13
Afraid of transmittable/contagious disease	n = 172	n = 105	n = 191	n = 158
Do not want to touch a stranger	n = 49	n = 22	n = 50	n = 24
Other reasons^d^	n = 50	n = 32	n = 36	n = 31
Unknown reason	n = 8	n = 19	n = 16	n = 4

a Including other reasons mentioned related to the open question: “I cannot do compressions with my wrist and hands”; “Rheumatoid arthritis”; “Panic”; “Paralysis”; “Shock”; “Fear”; “Discomfort”; “Uncertainty”.

b Including other reasons mentioned related to the open question: “I have severe cough”; “Blood”; “Vomiting”; “Unpleasant”; “Disgusting”; “Panic”; “State of shock”; “Fear”; “Discomfort”; “Uncomfortable”; “Uncertainty”; “Lack of practical skills”; “Compressions are most important”; “Depends on the condition of the person”.

c Including other reasons mentioned related to the open question: “I cannot do chest compressions because of my wrists and hands”; “Panic”; “Fear”; “Uncomfortable”; “Uncertainty”; “Shock”; “Lack of knowledge”; “I would probably want someone else to take the responsibility, If not — then I would”.

d Including other reasons mentioned related to the open question: “I have asthma”; “I have physical causes”; “Depends on the situation and the condition of the person”, “Blood”; “Vomiting”; “Mucus”; “Dirty”; “Open wound”; “Smells bad”; “Snuff”; “Unpleasant”; “Disgusting”; “Uncomfortable”; “Uneasiness about giving breaths to an unknown person”; “Little too intimate”; “A little scary to kiss an unknown person”; “Emotional causes”; “I would get nausea”; “Fear”; “Depends on whether it is a child or an old person”; “Stranger”; “Disgusting culture or religion”; “Drunk or drug affected”; “AIDS”; “Infection”; “Hygienic reasons”; “Shock”; “Panic”; “Do not want to do ventilations”; “With face protection I would do ventilations”; “With a pocket mask it would be more reasonable and safe”; “I might panic and forget the knowledge”; “I am afraid that I react like — I do not dare or want to, but I would like to respond and give both chest compressions and ventilations”; “Chest compressions are usually enough”; “The compressions are most important and whether I want to give ventilations depends on the situation”.

## 2 Experimental design, materials and methods

We conducted an analysis of data from an educational BLS project for workplaces in Sweden which were collected between 2014 and 2016. The design was experimental and compared two different types of practical training in BLS. Self-learning training constituted the experimental group and instructor-led training constituted the control group.

The study population was adult lay people with no previous BLS training or no BLS training within the past five years from 84 workplaces located in different places in the community, outside hospitals. The participants were cluster randomised to self-learning training or to instructor-led training in BLS. Detailed information on materials and methods is given in Bylow et al., 2019.

### 2.1 Statistical analysis

For the analysis of change from post-test directly after training to a retention test six months after training, only individuals participating on both occasions were included (n = 653 in the self-learning group and n = 521 in the instructor-led group). For dichotomous variables, we present the percentages of all available participants with discordant values on the two occasions, i.e. No+Yes (or criteria not fulfilled+criteria fulfilled) versus Yes+No (or criteria fulfilled+criteria not fulfilled), in the two training groups respectively. For continuous variables, we present the median of change from post-test to retention test, with corresponding 25th and 75th percentiles. P-values for change regarding dichotomous variables were calculated using Obuchowski's modified McNemar's test for clustered paired data [14], performed using the CLUSTPRO SAS macro [15], except when percentages were too small, when McNemar's exact test, without adjustment for clustering, was used. For changes in continuous variables, the Wilcoxon signed rank test for paired comparisons of clustered data [16] was used to calculate p-values. All tests are two-sided and p < 0.05 was considered statistically significant. SAS for Windows version 9.4 was used for all analyses performed.

## Financial support

Financial support is detailed in Ref. [1].

## Authors’ contributions

Authors’ contributions are detailed in Ref. [1].
