# Supplementary material for: Supplementary dataset to self-learning training compared with instructor-led training in basic life support
Source: Data Brief. 2019 May 26;25:104064. doi: 10.1016/j.dib.2019.104064 (PMC6600603; doi:10.1016/j.dib.2019.104064)
Supplement: Multimedia component 2 [file mmc2.docx]

Data statement

Additional data is available on request
