# Supplementary material for: Supplementary dataset to self-learning training compared with instructor-led training in basic life support
Source: Data Brief. 2019 May 26;25:104064. doi: 10.1016/j.dib.2019.104064 (PMC6600603; doi:10.1016/j.dib.2019.104064)
Supplement: Multimedia component 3 [file mmc3.doc]

**Meta-Data**

| ***Title:** | Supplementary dataset to self-learning training compared with instructor-led training in basic life support |
| --- | --- |
| ***Authors:** | Helene Bylow ^a^, Thomas Karlsson ^b^, Andreas Claesson ^c^, Margret Lepp ^d^, Jonny Lindqvist ^e^, Johan Herlitz ^a,e,f^ |
| ***Affiliations:** | ^a^ Department of Molecular and Clinical Medicine, Institute of Medicine, Sahlgrenska Academy, University of Gothenburg, Gothenburg, Sweden ^b^ Health Metrics Unit, Institute of Medicine, Sahlgrenska Academy, University of Gothenburg, Gothenburg, Sweden ^c^ Department of Medicine, Centre for Resuscitation Science, Karolinska Institute, Stockholm, Sweden ^d^ Institute of Health and Care Sciences, Sahlgrenska Academy, University of Gothenburg, Sweden; Østfold University College, Halden, Norway; School of Nursing and Midwifery, Griffith University, Australia ^e^ Centre of Registers Västra Götaland, Gothenburg, Sweden ^f^ Prehospen-Centre of Prehospital Research; Faculty of Caring Science, Work Life and Social Welfare; University of Borås, Sweden |
| ***Contact email:** | helene.bylow@gu.se |
| *****Co-authors**: | thomas.karlsson@gu.se  [johan.herlitz@hb.se](mailto:johan.herlitz@hb.se) [andreas.claesson@ki.se](mailto:andreas.claesson@ki.se) [margret.lepp@gu.se](mailto:margret.lepp@gu.se) [jonny.lindqvist@gu.se](mailto:jonny.lindqvist@gu.se) |
| ***CATEGORY:** | 182 Medicine (general) 251 Education |

**DATA IN BRIEF**

**Data Article**

**Title**Supplementary dataset to self-learning training compared with instructor-led training in basic life support

**Authors**Helene Bylow ^a^, Thomas Karlsson ^b^, Andreas Claesson ^c^, Margret Lepp ^d^, Jonny Lindqvist ^e^, Johan Herlitz ^a,e,f^

***Affiliations*** *^a^ Department of Molecular and Clinical Medicine, Institute of Medicine, Sahlgrenska Academy, University of Gothenburg, Gothenburg, Sweden
^b^ Health Metrics Unit, Institute of Medicine, Sahlgrenska Academy, University of Gothenburg, Gothenburg, Sweden
^c^ Department of Medicine, Centre for Resuscitation Science, Karolinska Institute, Stockholm, Sweden
^d^ Institute of Health and Care Sciences, Sahlgrenska Academy, University of Gothenburg, Sweden; Østfold University College, Halden, Norway; School of Nursing and Midwifery, Griffith University, Australia
^e^ Centre of Registers Västra Götaland, Gothenburg, Sweden
^f^ Prehospen-Centre of Prehospital Research; Faculty of Caring Science, Work Life and Social Welfare; University of Borås, Sweden*

**Contact email**: [helene.bylow@gu.se](mailto:helene.bylow@gu.se) (HB)

**Abstract**

In this article, we present supplementary data to the article entitled “Self-learning training versus instructor-led training in basic life support: a cluster randomised trial” [1]. In three supplementary files, we present the informed consent of the included participants, the modified instrument to calculate the total score for practical skills called “the Cardiff Test of basic life support and automated external defibrillation” and the questionnaire to obtain background factors, theoretical knowledge, self-assessed knowledge and confidence and willingness to act, distributed directly after training and six months after training. The results of comparisons between “directly after intervention” and “six months after intervention”, for each training group separately, are presented in three tables. We also present two tables showing the reasons why the participants were not prepared to perform compressions and/or ventilations in the event of a sudden out-of-hospital cardiac arrest.

**Specification table**

| Subject area | Medicine |
| --- | --- |
| More specific subject area | Cardiology, cardiac arrest and education |
| Type of data | Supplementary files and tables with analysed and raw data |
| How data were acquired | Data were acquired from an intervention with education in basic life support (BLS) and automated external defibrillation (AED). Informed consent was required from each participant. A modified instrument called “the Cardiff Test of basic life support and automated external defibrillation” calculated the total score for practical skills. A questionnaire directly after training and six months after training acquired background factors such as characteristics and theoretical knowledge, self-assessed knowledge, confidence and willingness. A training manikin connected to a data program measured the variables. A video camera filmed the assessment and was used to control the direct observation of the assessment. |
| Data format | Analysed and raw data |
| Experimental factors | This experiment was a cluster randomised, controlled trial. The self-learning training in BLS constituted the experimental group and the instructor-led training in BLS constituted the control group. The primary outcome was the total score on the Cardiff Test of basic life support and automated external defibrillation for adherence to the BLS-AED algorithm. |
| Experimental features | Participants were enrolled from a BLS-AED research project. Effectiveness of self-learning training in BLS-AED was compared with instructor-led training in BLS-AED. |
| Data source location | Data were collected at 84 workplaces in the south of Sweden in 2014 to 2016. Data source location: Alingsås, Almenäs, Borgstena, Borås, Brotorp, Brämhult, Byttorp, Dalsjöfors, Eskilsryd, Falköping, Fristad, Frufällan, Gothenburg, Helsingborg, Hyberg, Kinna, Kyllared, Limmared, Malmö, Mölnlycke, Sandared, Sjöbo, Sjömarken, Skene, Skövde, Spånga, Tranemo, Trollhättan, Viared and Viskafors. |
| Data accessibility | The data are presented in this article. |
| Related research article | Bylow H, Karlsson T, Claesson A, Lepp M, Lindqvist J, Herlitz J. Self-learning training versus instructor-led training for basic life support: A cluster randomised trial. Resuscitation. 2019. |

**Value of the data**

- **The data provide information on the informed consent, valuable for scientific researchers.**
- **The instrument called the Cardiff Test of basic life support and automated external defibrillation, modified to guidelines, was used to calculate the total score for practical skills and provide crucial information on the included variables. This can be valuable both for the interpretation of the data and for other experiments.**
- **The data provide the variables in the questionnaire directly after intervention and six months after intervention for an insight into the self-assessed variables and for further investigations.**
- **The data present changes from post-test to retention test, for each group separately, which may be useful for discussions, further insights and development of new experiments.**
- **The data provide reasons given by the participants for unwillingness to perform CPR in an OHCA situation, which may be useful for discussions, further insights and development of new experiments.**

**1. Data**

In this article, we present supplementary data to the article entitled “Self-learning training versus instructor-led training in basic life support: a cluster randomised trial”, where we compared the total score for practical skills and theoretical and self-assessed knowledge, confidence and willingness to act, between two training interventions in basic life support (BLS) with automated external defibrillation (AED) [1]. The training was based on the European Resuscitation Council (ERC) guidelines [2, 3]. Education with frequent training in BLS with AED may increase early high-quality cardiopulmonary resuscitation (CPR) with early defibrillation in the event of a sudden out-of-hospital cardiac arrest (OHCA) [4].

The data shared in this article contain supplementary files and tables and supplement the article by Bylow et al., 2019. Informed consent was required from each participant and is presented in this article in Supplementary file 1.

The instrument used to calculate the total score, the modified Cardiff Test of basic life support and external defibrillation, is presented in Supplementary file 2. Actions to evaluate education in BLS and the performance of CPR with an AED, definitions and scoring documents to assess the participants, were introduced as a statement on the uniform reporting of education in resuscitation [5, 6], in 2003. The Cardiff Test in this article was based on the previous statement and modified according to the European Resuscitation Council (ERC) guidelines [2, 3] and previous studies [5-12] and was then tested in a pilot study [13].

The questionnaire used to collect background factors and the participants’ self-assessed theoretical knowledge, confidence and willingness to perform CPR with an AED in a real-life OHCA situation, directly after intervention and six months later, is presented in Supplementary file 3.

To supplement the cluster randomised, controlled trial [1], we present data from comparisons between post-test and retention test, for each training group, i.e. self-learning training and instructor-led training in BLS, separately, in this article. Table 1 presents analysed values from the Cardiff Test of basic life support and external defibrillation, Table 2 shows analysed values from individual variables and Table 3 presents analysed values from the self-assessed variables.

In Table 4, we present reasons given by the participants for not being willing to perform compressions and/or ventilations in a sudden real-life out-of-hospital cardiac situation.

**2. Experimental design, materials and methods**

We conducted an analysis of data from an educational BLS project for workplaces in Sweden which were collected between 2014 to 2016. The design was experimental and compared two different types of practical training in BLS. Self-learning training constituted the experimental group and instructor-led training constituted the control group.

The study population was adult lay people with no previous BLS training or no BLS training within the past five years from 84 workplaces located in different places in the community, outside hospitals. The participants were cluster randomised to self-learning training or to instructor-led training in BLS. Detailed information on materials and methods is given in Bylow et al., 2019.

**2.1 Statistical analysis**

For the analysis of change from post-test directly after training to a retention test six months after training, only individuals participating on both occasions were included (n=653 in the self-learning group and n=521 in the instructor-led group). For dichotomous variables, we present the percentages of all available participants with discordant values on the two occasions, i.e. No+Yes (or criteria not fulfilled+criteria fulfilled) versus Yes+No (or criteria fulfilled+criteria not fulfilled), in the two training groups respectively. For continuous variables, we present the median of change from post-test to retention test, with corresponding 25^th^ and 75^th^ percentiles. P-values for change regarding dichotomous variables were calculated using Obuchowski’s modified McNemar’s test for clustered paired data [14], performed using the CLUSTPRO SAS macro [15], except when percentages were too small, when McNemar’s exact test, without adjustment for clustering, was used. For changes in continuous variables, the Wilcoxon signed rank test for paired comparisons of clustered data [16] was used to calculate p-values. All tests are two-sided and p < 0.05 was considered statistically significant. SAS for Windows version 9.4 was used for all analyses performed.

**Acknowledgments**

Acknowledgements are detailed in Ref. [1]

**Financial support**

Financial support is detailed in Ref. [1]

**Authors’ contributions**

Authors’ contributions are detailed in Ref. [1]

**References**
