# Supplementary material for: Supplementary dataset to self-learning training compared with instructor-led training in basic life support
Source: Data Brief. 2019 May 26;25:104064. doi: 10.1016/j.dib.2019.104064 (PMC6600603; doi:10.1016/j.dib.2019.104064)
Supplement: Multimedia component 5 [file mmc5.docx]

***Supplementary file 2***

Description of the modified version of the Cardiff Test of basic life support and automated external defibrillation (Cardiff Test) Supplementary file to the article, Data supplementary to the article; self-learning training compared with instructor-led training in basic life support. This version of Cardiff Test is based on the European Resuscitation Council Guidelines, 2010.

The test lasted for three minutes in order optimally to identify the cardiac arrest for no more than 30 seconds, call for help and perform cardiopulmonary resuscitation (CPR) and about two minutes for using the automated external defibrillator (AED). The AED was delivered three minutes after the scenario start. The test was terminated after one shock and the resumption of CPR. The practical part was filmed by a mounted visible video camera on the wall after personal information and a signed consent form by the participant. The test was started after a simulated sudden out-of-hospital cardiac arrest (OHCA) scenario was described by the assessor to the participant who was sitting in a chair in a regular living room beside a dressed manikin, lying on the floor.

*“You are at work. A colleague looks pale, puts her/his hand in the middle of the her/his chest and says, I have chest pain and then suddenly collapses in front of you. Act as if it was a real-life situation!”*

The environment was as close as possible to a real-life situation and equally for all assessments. The Cardiff Test score sheet was marked manually from direct observation, registrations from the Laerdal PC Skill Reporting System (Laerdal Medical, Stavanger, Norway) and checked by the film after the test. All the variables were then entered into a database system.

| **Modified version of the Cardiff Test of basic life support and external defibrillation** | |
| --- | --- |
| **Description of variables in the Cardiff Test**  Maximum score 70 — minimum score 19 | **Points**  (70-19) |
| **1. Checks responsiveness—by talking**  2 points: Yes  1 point: No  **Description: Responsiveness—talk**  The participant must check responsiveness by some verbal communication, talking, saying “Are you all right”, for example. If performed, a yes was marked, if not, a no was marked by direct observation and registration on the Cardiff Test score sheet. | **(2-1)** |
| **2. Checks responsiveness—by shaking**  3 points: Yes  2 points: No  1 point: Potentially dangerous  **Description: Responsiveness—shake**  The participant must check responsiveness by gently shaking the victim’s shoulders. If performed, a yes was marked, if not, a no was marked and, if the shaking was violent, potentially causing injuries, the lowest score was marked by direct observation and registration on the Cardiff Test. | **(3-1)** |
| **3. Opens airway—head tilt and chin lift**  5 points: Perfect as instructed  4 points: Acceptable  3 points: Attempted other  2 points: Attempted visible but fails or only one element  1 point: No  **Description: Open airway—head tilt and chin lift**  For perfect as instructed, the participant must clearly and visibly place one hand on the victim’s forehead, tilt the head back and lift the chin with the fingertips on the hard bone to open the airway. For acceptable the manoeuvre must open the airway as instructed, close to perfect. If attempting technique other than that described by ERC guidelines, it must be similar to as instructed. If the participant failed or only used one element, the next lowest score was marked. No was marked when no attempt to open the airway was made at all. Direct observation and registration on the Cardiff Test were used even if the PC Skill Reporting System also registers it. | **(5-1)** |
| **4. Checks breathing—look, listen and feel**  2 points: Yes  1 point: No  **Description: Breathing—look, listen and feel**  Yes, was marked if the participant performed open airway together with look, listen and feel correctly and for no more than ten seconds. No was marked if no attempt to check breathing was made. Direct observation, a stopwatch and registration on the Cardiff Test were used. | **(2-1)** |
| **5. Calls 112—or asks for help calling 112**  2 points: Yes  1 point: No  **Description: Call 112**  For yes, the participant had to call to 112 or ask someone else to call 112 (the national emergency number) within the first minute from the scenario start. If not, no was marked on the Cardiff Test using direct observation, a stopwatch and registration on the Cardiff Test. | **(2-1)** |
| **6. Send someone for AED**  2 points: Yes  1 point: No  **Description: Send for AED**  For yes, the participant had to ask someone to bring the AED within three minutes. If not, no was marked. Direct observation, a stopwatch and registration on the Cardiff Test were used. | **(2-1)** |
| **7. Starts CPR—compression/ventilation ratio**  4 points: 30:2 (28-32:2)  3 points: Another ratio  2 points: Compressions only  1 point: Ventilations only  **Description: Start CPR**  For the highest score, the participant had to start with thirty compressions (28-32 were accepted), two ventilations and then continue the ratio 30:2. Some misses in the ratio were accepted, such as 28-32 for compressions and failed ventilations. Another ratio was marked if a different ratio from 28-32:2 was used, for example, 2:30. Compressions and ventilations only were marked if the participant intended to perform only one of them. For the CPR ratio, we used 30:2 for six points, as the maximum according to ERC guidelines for trained, five points for another ratio, as the national education teaches both compressions and ventilations, and three points for compressions only, as this was an educational intervention. Direct observation, registration from the Laerdal PC Skill Reporting System were used directly and transferred after the test to the Cardiff Test score sheet. The time from scenario start to first compression was counted by a stopwatch and from the PC Skill Reporting System, marked on the Cardiff Test score sheet. | **(4-1)** |
| **8. Hand placement compressions**  4 points: Correct  3 points: Other wrong  2 points: Too low  1 point: Not attempted  **Description: Hand placement for compressions**  The correct hand position according to previous validated Cardiff Test means that all compressions must be correct for the highest score. They must all be placed according to ERC guidelines, with the heel of one hand on the centre of the victim’s chest, on the lower half of the breast bone, the heel of the other hand on top of the first hand and with interlocked fingers. Wrong was recorded for compressions too high up on the breast bone, to the right or to the left of the breast bone, if the participant used too much of the hand or had very large hands or used his/her fist. Too low compressions were recorded if just one compression was too low on the breast bone. If no compressions were performed, not attempted was marked. Direct observation, registration from the Laerdal PC Skill Reporting System were used and transferred to the Cardiff Test score sheet. | **(4-1)** |
| **9. Average compression depth**  6 points: 50-59 mm  5 points: ≥ 60 mm  4 points: 35-49 mm  2 points: < 35  1 point: Not attempted  **Description: Average compression depth**  To adjust for current guidelines on compression depth and keep the score as in validated previous studies with six points, we had to remove three points from the score. The PC Skill Reporting System version, 2.4.1, only measured up to 60 mm and, for this reason, we decided to score 50-59 mm. Those who performed compressions of 60 mm received the next highest score. Direct observation, registration from the Laerdal PC Skill Reporting System were used and transferred to the Cardiff Test score sheet. | **(6-1)** |
| **10 Average compression rate**  6 points: 100-120  5 points: 121-140  4 points: 80-99  3 points: > 140  2 points: < 80  1 point: Not attempted  **Description: Average compression rate**  The six-level scoring scale was kept for the compression rate as previous validated studies. We used the same level as compression depth to be consistent. Both the exact value for the average compression rate from the Laerdal PC Skill Reporting System and the Cardiff Test points were noted on the sheet. | **(6-1)** |
| **11. Total compressions counted**  6 points: 140-190  5 points: > 190  4 points: 121-139  3 points: 81-120  2 points: 1-80  1 point: Not attempted  **Description: Total compressions counted**  Total compressions were counted for three minutes. This was the time from the scenario start to when the AED was delivered to the participant. The time included for optimally identifying the cardiac arrest, calling for help, asking for an AED, compressions and ventilations. The total number of counted compressions are therefore dependent on how much time the participant took for each step. Data from the Laerdal PC Skill Reporting System were transferred to the Cardiff Test score sheet both as the exact value and as the Cardiff Test points. | **(6-1)** |
| **12. Average ventilation volume**  5 points: 500-600 ml  4 points: 1-499 ml  3 points: > 600 ml  2 points: 0 ml  1 point: Not attempted  **Description: Average ventilation volume**  Like previous validated studies, we kept the five-level scale for ventilation volume. Two points was marked if the participant attempted ventilations but failed, the chest did not rise and no volume was registered in the Laerdal PC Skill Reporting System. For one point, ventilations were not performed at all. Direct observation, registration from the Laerdal PC Skill Reporting System were used directly and transferred after the test for scoring to the Cardiff Test score sheet, both as the exact value and as the Cardiff Test points. | **(5-1)** |
| **13. Total ventilations counted**  5 points: 8-12  4 points: 1-7  3 points: > 12  2 points: 0  1 point: Not attempted  **Description: Total ventilations counted**  Total ventilations were counted for three minutes. This was the time from the scenario start to when the AED was delivered to the participant. The time included for optimally identifying the cardiac arrest, calling for help, asking for an AED, compressions and ventilations. The total number of counted ventilations are therefore dependent on how much time the participant took for each step. Direct observation, registration from the Laerdal PC Skill Reporting System were used directly and transferred after the test for scoring to the Cardiff Test score sheet, both as an exact value and as the Cardiff Test points. | **(5-1)** |
| **14. Total hands-off time**  4 points: ≤ 60 second  3 points: 61-90 seconds  2 points: 91-135 seconds  1 points: > 135 seconds  **Description: Total hands-off time**  When no compressions were performed in the scenario, the total hands-off time was measured from the Laerdal PC Skill Reporting System. The data was transferred to the Cardiff Test scoring sheet, both as an exact value and as the Cardiff Test point. | **(4-1)** |
| **15. Switch on AED**  2 points: Yes  1 point: No  **Description: Switch on AED**  After three minutes from the scenario start, the assessor placed the AED next to the manikin and started the stopwatch. If the participant switched the AED on, a yes was marked. The time for delivering the AED was calculated for a workplace environment with an onsite AED and for enough time to measure CPR and the decision to use the AED or not. Direct observation with registration was used directly and transferred to the Cardiff Test score sheet. | **(2-1)** |
| **16. Attach electrode pads**  6 points: Both electrodes completely in areas  5 points: One electrode completely in area and one crossing the border of area  4 points: One electrode completely in area and one electrode outside the area  3 points: Both electrodes crossing the area  2 points: Both electrodes outside the areas  1 point: Electrodes not attached or plugged into AED  **Description: Attach electrode pads**  Previous studies with pictures were used for the scoring system for the correct position for the electrode pads on the victim’s bare chest. Direct observation with registration was used directly and transferred to the Cardiff Test score sheet. | **(6-1)** |
| **17. Checks safety before shocking**  2 points: Yes, performed  1 point: No, not performed  **Description: Checks safety of bystanders and him/herself before shocking**  The participant must ensure that nobody or him/herself is in contact with the victim using a verbal and visual check and communicating, for example, with “stand clear”, before the shock button is pushed. No was marked if the participant did not check for safety. | **(2-1)** |
| **18. Deliver shock as directed from the AED**  2 points: Yes  1 point: No  The time from when the AED was placed next to the manikin to the first shock was counted using a stopwatch and marked on the Cardiff Test score sheet. | **(2-1)** |
| **19. Continues CPR directly after shocking, as directed from the AED**  2 points: Yes  1 point: No  **Description: Continues CPR directly after shocking**  After shocking, the voice prompt from the AED told the participant to continue CPR. Direct observation with registration was used directly and transferred to the Cardiff Test score sheet. | **(2-1)** |
| **Maximum points 70**  **Minimum points 19** | **(70-19)** |
