# Supplementary material for: Supplementary dataset to self-learning training compared with instructor-led training in basic life support
Source: Data Brief. 2019 May 26;25:104064. doi: 10.1016/j.dib.2019.104064 (PMC6600603; doi:10.1016/j.dib.2019.104064)
Supplement: Multimedia component 6 [file mmc6.docx]

***Supplementary file 3***

Supplementary file 3 to the data article: Data supplementary to self-learning training compared with instructor-led training in basic life support. Questionnaire for all study participants in the BLS project 2014-2016, directly after intervention relating to education in basic life support.

*ONE response option to each question. When SEVERAL options are available, this is indicated. When no options are presented, the question is open ended and the respondent is free to write as much as he/she likes.*

QUESTIONNAIRE DIRECTLY AFTER INTERVENTION

BACKGROUND FACTORS

1) Participant, personal code number:

2) Educational intervention; year, month, day and time:

3) Follow-up, directly after intervention; year, month, day and time:

BACKGROUND FACTOR PREVIOUS PRACTICAL TRAINING IN BLS, CPR and AED

4) Have you previously trained in:

a) chest compressions? Yes < 5 years ago/Yes > than 5 years ago/No, never

b) ventilations? Yes < 5 years ago/Yes > than 5 years ago/No, never

c) automated external defibrillator? Yes < 5 years ago/Yes > than 5 years ago/No, never

BACKGROUND FACTORS ON PREVIOUSLY EXPERIENCED REAL-LIFE SITUATIONS

5) Have you ever been involved when someone suffered a:

a) suspected stroke? Yes/No/Do not know

b) suspected heart attack (acute myocardial infarction)? Yes/No/Do not know

c) suspected sudden cardiac arrest? Yes/No/Do not know

SELF-ASSESSED IMPORTANCE OF BLS LEARNING

6) Do you think it is important to learn basic life support, cardiopulmonary resuscitation and to use an automated external defibrillator? Yes/No/Do not know

SELF-ASSESSED THEORETICAL AND PRACTICAL KNOWLEDGE

7) Do you think your theoretical knowledge and practical skills are sufficient to perform:

a) chest compressions? Yes/No/Do not know

b) ventilations? Yes/No/Do not know

c) instructions from an automated external defibrillator? Yes/No/Do not know

SELF-ASSESSED CONFIDENCE

8) Do you feel more confident after the intervention than before, to intervene and act in a real-life sudden cardiac arrest situation? Yes/No/Do not know

SELF-ASSESSED WILLINGNESS TO ACT AND START CPR – relative

9) Imagine a situation at home. How do you think you would act if a close friend or a relative suffered a sudden cardiac arrest?

I would not dare or want to intervene or act

I would perform chest compressions, only

I would perform ventilations, only

I would perform both chest compressions and ventilations

10) If you answered that you would NOT dare or want to perform chest compressions, indicate possible reasons why: (SEVERAL options are possible)

a) Lack of knowledge

b) Afraid of hurting the person

c) Afraid of transmissible/contagious disease

d) Other reasons

If other reasons, describe which (*open ended)*:

11) If you answered that you would NOT dare or want to perform ventilations, indicate possible reasons why: (SEVERAL options are possible)

a) Lack of knowledge

b) Afraid of hurting the person

c) Afraid of transmissible/contagious disease

d) Other reasons

If other reasons, describe which (*open ended)*:

SELF-ASSESSED WILLINGNESS TO ACT AND START CPR – unknown person

12) Imagine a situation at a bus stop. How do you think you would act if an unknown person suffered a sudden cardiac arrest?

I would not dare or want to intervene or act

I would perform chest compressions, only

I would perform ventilations, only

I would perform both chest compressions and ventilations

13) If you answered that you would NOT dare or want to perform chest compressions, indicate possible reasons why: (SEVERAL options are possible)

a) Lack of knowledge

b) Afraid of hurting the person

c) Afraid of transmissible/contagious disease

d) Do not want to touch a stranger

e) Other reasons

If other reasons, describe which (*open ended)*:

14) If you answered that you would NOT dare or want to perform ventilations, indicate possible reasons why: (SEVERAL options are possible)

a) Lack of knowledge

b) Afraid of hurting the person

c) Afraid of transmissible/contagious disease

d) Do not want to touch a stranger

e) Other reasons

If other reasons, describe which (*open ended)*:

SELF-ASSESSED WILLINGNESS TO USE AN AED

15) Imagine a situation at work. How do you think you would act in a situation in which your colleague was performing cardiopulmonary resuscitation, the ambulance was not yet present and the workplace had an automated external defibrillator on site? Would you use the automated external defibrillator? Yes/No/Do not know

16) If you answered that you would not dare or want to use the automated external defibrillator, indicate possible reasons why: (SEVERAL options are possible)

a) Lack of knowledge

b) Afraid of hurting the person

c) Other reasons

If other reasons, describe which (*open ended)*:

SELF-ESTIMATED EXPERIENCED LEARNING FACTORS FOR PRACTICAL SKILLS DEPENDENT ON THE EDUCATION

17) If the education made you feel that you can start cardiopulmonary resuscitation and use an automated external defibrillator, what was it in the education that specifically contributed (*open ended)*: ­­­­­­­­­­­­­­­­­­­­­­­­

­­­­­­­­­­­­­­­­­­­­­­­­­­­­­­­­­­­­­­­­­­­­­­­

SELF-ESTIMATED MISSING LEARNING FACTORS FOR PRACTICAL SKILLS DEPENDENT ON THE EDUCATION

18) If the education did NOT make you feel that you could start cardiopulmonary resuscitation and use an automated external defibrillator, what would have been needed for you to intervene and feel that you could start cardiopulmonary resuscitation and use an automated external defibrillator (*open ended)*:

STROKE SYMPTOMS
19) Which symptoms often occur, regarding a stroke?

a) Pain on one side of the body: Yes/No/Do not know

b) Pain on both the left and the right side of the body: Yes/No/Do not know

c) Weakness on one side of the body: Yes/No/Do not know

d) Weakness on both the left and the right side of the body: Yes/No/Do not know

e) Symptoms occur slowly: Yes/No/Do not know

f) Symptoms occur quickly: Yes/No/Do not know

g) Difficulty speaking or slurred speech: Yes/No/Do not know

ACUTE MYOCARDIAL INFARCTION SYMPTOMS

20) What symptoms often occur, regarding a heart attack (acute myocardial infarction)?

a) Discomfort or pain in the right arm: Yes/No/Do not know

b) Discomfort or pain in the left arm: Yes/No/Do not know

c) Discomfort or pain in the chest: Yes/No/Do not know

d) Discomfort or pain in the right leg: Yes/No/Do not know

e) Discomfort or pain in the left leg: Yes/No/Do not know

f) Discomfort or pain in the back: Yes/No/Do not know

g) Discomfort or pain in the stomach: Yes/No/Do not know

h) Headache: Yes/No/Do not know

i) Nausea: Yes/No/Do not know

THEORETICAL KNOWLEDGE OF FIRST ACTION IF STROKE OR ACUTE MYOCARDIAL INFARCTION

21) If symptoms of a stroke or heart attack (acute myocardial infarction) occur, what is your first action?

Call the medical on-call service, 1177

Call the health centre

Call the emergency services, 112

Wait fifteen minutes to see if the symptoms disappear

Do not know

THEORETICAL KNOWLEDGE OF FIRST ACTION IF CARDIAC ARREST

22) What is your first action if you find a person with cardiac arrest?

Start cardiopulmonary resuscitation

Find and bring the nearest automated external defibrillator

Place the victim in the recovery position

Call 112

Do not know

THEORETICAL KNOWLEDGE OF HEALTHY LIFE STYLE FACTORS

23) Which of the following living habits are regarded as healthy lifestyle factors?

a) Regular physical exercise: Yes/No/Do not know

b) Smoking: Yes/No/Do not know

c) Eating fruit and vegetables daily: Yes/No/Do not know

d) Being mostly sedentary daily: Yes/No/Do not know

e) Eating fish two or three times a week: Yes/No/Do not know

f) Daily exercise such as walking or cycling: Yes/No/Do not know

BACKGROUND FACTOR LANGUAGE

24) Is your mother tongue (the first language you learned) Swedish? Yes/No/Do not know

If NO, do you consider that you are able to read and understand Swedish without difficulty? Yes/No/Do not know

BACKGROUND FACTOR CVD

25) Do you have any known cardiovascular disease? Yes/No/Do not know

If YES, can you briefly describe the kind of disease (*open ended)*:

BACKGROUND FACTOR CVD

26) Have any close relatives or other related person any known cardiovascular disease? Yes/No/Do not know?

If YES, can you briefly describe the kind of disease (*open ended)*:

BACKGROUND FACTOR EDUCATION

27) What is your highest level of education?

a) Have not attended school

b) Primary/elementary school (about age 6-16)

c) Secondary/high school (about age 17-20)

d) College/university (from about age 21)

BACKGROUND FACTOR OCCUPATION

28) Occupation:

BACKGROUND FACTOR AGE

29) Age:

BACKGROUND FACTOR GENDER

30) Gender:

BACKGROUND FACTOR WEIGHT

31) Weight:

BACKGROUND FACTOR HEIGHT

32) Height:

OVERALL COMMENTS

33) Other comments (*open ended)*:

Supplementary file 3 to the data article: Data supplementary to self-learning training compared with instructor-led training in basic life support. Questionnaire for all study participants in the BLS project 2014-2016, six months after intervention relating to education in basic life support.

*ONE response option to each question. When SEVERAL options are available, this is indicated. When no options are presented, the question is open ended and the respondent is free to write as much as he/she likes.*

**QUESTIONNAIRE SIX MONTHS AFTER INTERVENTION**

**BACKGROUND FACTORS**

1) Participant, personal code number

2) Educational intervention; year, month, day and time

3) Follow-up, six months after intervention; year, month day and time

**THEORETICAL AND PRACTICAL BENEFITS OF THE INTERVENTION**

4) After the intervention, have you experienced theoretical or practical benefits from the education on:

a) chest compressions? Yes/No/Do not know

b) ventilations? Yes/No/Do not know

c) automated external defibrillator? Yes/No/Do not know

If yes, describe how (o*pen ended)*:

**BACKGROUND FACTORS ON PREVIOUSLY EXPERIENCED REAL-LIFE SITUATIONS**

5) After the intervention, have you been involved when someone suffered a:

a) suspected stroke? Yes/No/Do not know

b) suspected heart attack (acute myocardial infarction)? Yes/No/Do not know

c) suspected sudden cardiac arrest? Yes/No/Do not know

**SELF-ASSESSED IMPORTANCE OF BLS LEARNING**

6) Do you think it is important to learn basic life support, cardiopulmonary resuscitation and to use an automated external defibrillator? Yes/No/Do not know

**SELF-ASSESSED THEORETICAL AND PRACTICAL KNOWLEDGE**

7) Do you think your theoretical knowledge and practical skills are sufficient to perform:

a) chest compressions? Yes/No/Do not know

b) ventilations? Yes/No/Do not know

c) instructions from an automated external defibrillator? Yes/No/Do not know

**SELF-ASSESSED CONFIDENCE**

8) Do you feel more confident after the intervention than before, to intervene and act in a real-life sudden cardiac arrest situation? Yes/No/Do not know

**SELF-ASSESSED WILLINGNESS TO ACT AND START CPR – relative**

9) Imagine a situation at home. How do you think you would act if a close friend or a relative suffered a sudden cardiac arrest?

I would not dare or want to intervene or act

I would perform chest compressions, only

I would perform ventilations, only

I would perform both chest compressions and ventilations

10) If you answered that you would NOT dare or want to perform chest compressions, indicate possible reasons why: (SEVERAL options are possible)

a) Lack of knowledge

b) Afraid of hurting the person

c) Afraid of transmissible/contagious disease

d) Other reasons

If other reasons, describe which (*open ended)*:

11) If you answered that you would NOT dare or want to perform ventilations, indicate possible reasons why: (SEVERAL options are possible)

a) Lack of knowledge

b) Afraid of hurting the person

c) Afraid of transmissible/contagious disease

d) Other reasons

If other reasons, describe which (*open ended)*:

**SELF-ASSESSED WILLINGNESS TO ACT AND START CPR – unknown person**

12) Imagine a situation at a bus stop. How do you think you would act if an unknown person suffered a sudden cardiac arrest?

I would not dare or want to intervene or act

I would perform chest compressions, only

I would perform ventilations, only

I would perform both chest compressions and ventilations

13) If you answered that you would NOT dare or want to perform ventilations, indicate possible reasons why: (SEVERAL options are possible)

a) Lack of knowledge

b) Afraid of hurting the person

c) Afraid of transmissible/contagious disease

d) Do not want to touch a stranger

e) Other reasons

If other reasons, describe which (*open ended)*:

14) If you answered that you would NOT dare or want to perform chest compressions, indicate possible reasons why: (SEVERAL options are possible)

a) Lack of knowledge

b) Afraid of hurting the person

c) Afraid of transmissible/contagious disease

d) Do not want to touch a stranger

e) Other reasons

If other reasons, describe which (*open ended)*:

**SELF-ASSESSED WILLINGNESS TO USE AN AED**

15) Imagine a situation at work. How do you think you would act in a situation when your colleague was performing cardiopulmonary resuscitation, the ambulance was not yet present and the workplace had an automated external defibrillator on site? Would you use the automated external defibrillator? Yes/No/Do not know

16) If you answered that you would NOT dare or want to use the automated external defibrillator, indicate possible reasons why: (SEVERAL options are possible)

a) Lack of knowledge

b) Afraid of hurting the person

c) Other reasons

If other reasons, describe which (*open ended)*:

**SELF-ESTIMATED EXPERIENCED LEARNING FACTORS FOR PRACTICAL SKILLS DEPENDENT ON THE EDUCATION**

17) If the education made you feel that you can start cardiopulmonary resuscitation and use an automated external defibrillator, what was it in the education that specifically contributed (*open ended)*: ­­­­­­­­­­­­­­­­­­­­­­­­

­­­­­­­­­­­­­­­­­­­­­­­­­­­­­­­­­­­­­­­­­­­­­­­

**SELF-ESTIMATED MISSING LEARNING FACTORS FOR PRACTICAL SKILLS DEPENDENT ON THE EDUCATION**

18) If the education did NOT make you feel that you could start cardiopulmonary resuscitation and use an automated external defibrillator, what would have been needed for you to intervene and feel that you could start cardiopulmonary resuscitation and use an automated external defibrillator (*open ended)*:

**STROKE SYMPTOMS**
19) Which symptoms often occur, regarding a stroke?

a) Pain on one side of the body: Yes/No/Do not know

b) Pain on both the left and the right side of the body: Yes/No/Do not know

**c) Weakness on one side of the body: Yes/**No/Do not know

d) Weakness on both the left and the right side of the body: Yes/No/Do not know

e) Symptoms occur slowly: Yes/No/Do not know

**f) Symptoms occur quickly:** **Yes**/No/Do not know

**g) Difficulty speaking or slurred speech: Yes**/No/Do not know

**ACUTE MYOCARDIAL INFARCTION SYMPTOMS**

20) What symptoms often occur, regarding a heart attack (acute myocardial infarction)?

**a) Discomfort or pain in the right arm: Yes**/No/Do not know

**b) Discomfort or pain in the left arm: Yes**/No/Do not know

**c) Discomfort or pain in the chest: Yes**/No/Do not know

d) Discomfort or pain in the right leg: Yes/No/Do not know

e) Discomfort or pain in the left leg: Yes/No/Do not know

**f) Discomfort or pain in the back: Yes**/No/Do not know

**g) Discomfort or pain in the stomach: Yes**/No/Do not know

h) Headache: Yes/No/Do not know

**i) Nausea: Yes/**No/Do not know

**THEORETICAL KNOWLEDGE OF FIRST ACTION IF STROKE OR ACUTE MYOCARDIAL INFARCTION**

21) If symptoms of a stroke or heart attack (acute myocardial infarction) occur, what is your **first** action?

Call the medical on-call service, 1177

Call the health centre

**Call the emergency services, 112**

Wait fifteen minutes to see if the symptoms disappear

Do not know

**THEORETICAL KNOWLEDGE OF FIRST ACTION IF CARDIAC ARREST**

22) What is your **first** action if you find a person with cardiac arrest?

Start cardiopulmonary resuscitation

Find and bring the nearest automated external defibrillator

Place the victim in the recovery position

**Call 112**

Do not know

**THEORETICAL KNOWLEDGE OF HEALTHY LIFE STYLE FACTORS**

23) Which of the following living habits are regarded as healthy lifestyle factors?

**a) Regular physical exercise: Yes**/No/Do not know

b) Smoking: Yes/No/Do not know

**c) Eating fruit and vegetables daily: Yes**/No/Do not know

d) Being mostly sedentary daily: Yes/No/Do not know

**e) Eating fish two or three times a week: Yes**/No/Do not know

**f) Daily exercise such as walking or cycling: Yes**/No/Do not know

**BACKGROUND FACTOR LANGUAGE**

24) Is your mother tongue (the first language you learned) Swedish? Yes/No/Do not know

If NO, do you consider that you are able to read and understand Swedish without difficulty? Yes/No/Do not know

**BACKGROUND FACTOR CVD**

25) Do you have any known cardiovascular disease? Yes/No/Do not know

If YES, can you briefly describe the kind of disease (*open ended)*:

**BACKGROUND FACTOR CVD**

26) Have any close relatives or other related person any known cardiovascular disease? Yes/No/Do not know?

If YES, can you briefly describe the kind of disease (*open ended)*:

**BACKGROUND FACTOR EDUCATION**

27) What is your highest level of education?

a) Have not attended school

b) Primary/elementary school (about age 6-16)

c) Secondary/high school (about age 17-20)

d) College/university (from about age 21)

**BACKGROUND FACTOR OCCUPATION**

28) Occupation:

**BACKGROUND FACTOR AGE**29) Age:

**BACKGROUND FACTOR GENDER**30) Gender:

**BACKGROUND FACTOR WEIGHT**

31) Weight:

**BACKGROUND FACTOR HEIGHT**

32) Height:

**BACKGROUND FACTOR OTHER SOURCES OF INFORMATION**

33) Have you completed the web-based education called Help-Brain-Heart?

No, never
 Yes, once

Yes, several times

Do not know

If YES, what do you think about it (*open ended)*:

**BACKGROUND FACTOR OTHER SOURCES OF INFORMATION**

34) Have you recommended the web-based education called Help-Brain-Heart to someone else and, if yes, who (for example relatives or friends):

**BACKGROUND FACTOR OTHER SOURCES OF INFORMATION**

35) Have you looked at and read the mobile application called Rädda hjärtat (Save the heart):

No, never

Yes, once

Yes, several times

Do not know

If YES, what do you think about it (*open ended)*:

36) Have you recommended the mobile application named Rädda hjärtat (Save the heart) to someone else and, if yes, who (for example relatives or friends) (*open ended)*:

**SELF-ESTIMATED EXPECTED EXPERIENCE TO INTERVENE**

37) What would it be like for you to intervene in a real-life acute situation? How do you think? How do you feel? (*open ended):*

**SELF-REPORTED OVERALL EXPERIENCE OF THE INTERVENTION**

38) Here, you can make other comments on your overall experience of the education (*open ended)*:
