# Supplementary material for: Supplementary dataset to self-learning training compared with instructor-led training in basic life support
Source: Data Brief. 2019 May 26;25:104064. doi: 10.1016/j.dib.2019.104064 (PMC6600603; doi:10.1016/j.dib.2019.104064)
Supplement: Multimedia component 1 [file mmc1.docx]

**Dear Editor and Co-Editors of the journal Data in Brief,**

The authors declare that they have no conflicts of interests.

Sincerely, on behalf of the authors,

Helene Bylow

Corresponding author

**Authors**
Helene Bylow ^a^, Thomas Karlsson ^b^, Andreas Claesson ^c^, Margret Lepp ^d^, Jonny Lindqvist ^e^, Johan Herlitz ^a,e,f^

***Affiliations***
 *^a^ Department of Molecular and Clinical Medicine, Institute of Medicine, Sahlgrenska Academy, University of Gothenburg, Gothenburg, Sweden
^b^ Health Metrics Unit, Institute of Medicine, Sahlgrenska Academy, University of Gothenburg, Gothenburg, Sweden
^c^ Department of Medicine, Centre for Resuscitation Science, Karolinska Institute, Stockholm, Sweden
^d^ Institute of Health and Care Sciences, Sahlgrenska Academy, University of Gothenburg, Sweden; Østfold University College, Halden, Norway; School of Nursing and Midwifery, Griffith University, Australia
^e^ Centre of Registers Västra Götaland, Gothenburg, Sweden
^f^ Prehospen-Centre of Prehospital Research; Faculty of Caring Science, Work Life and Social Welfare; University of Borås, Sweden*
